# Prediction of Gut Wall Integrity Loss in Viral Gastroenteritis by Non-Invasive Marker

**DOI:** 10.3889/oamjms.2015.023

**Published:** 2015-03-05

**Authors:** Hala G. Elnady, Lobna S. Sherif, Maysa T. Saleh, Inas R. El-Alameey, Mai M. Youssef, Amal I. El Shafie, Iman Helwa, Haiam Abdel Raouf, Ahmed N. EL-Taweel

**Affiliations:** 1 *Child Health Department, National Research Centre, Cairo, Egypt*; 2 *Health Radiation Research Department, National Centre for Radiation Research and Technology, Cairo, Egypt*; 3 *Immunogenetics Department, National Research Centre, Cairo, Egypt*; 4 *Environment Virology Laboratory and Water Pollution Department, National Research Centre, Cairo, Egypt*

**Keywords:** Viral Gastroenteritis, Gut Wall Integrity Loss, Non Invasive Marker, Predicition

## Abstract

**BACKGROUND:**

Intestinal fatty acid binding proteins (I-FABPs) are mainly expressed in the intestinal villi, which are the initial site of destruction in viral gastroenteritis.

**AIM:**

This study was designed to assess serum I-FABPs as a predictor of gut wall integrity loss in viral gastroenteritis.

**PATIENTS AND METHODS:**

This case-control cross-sectional study was conducted on 93 cases of acute viral gastroenteritis. Twenty-eight healthy children matching in age were recruited as control group. Serum I-FABPs were measured using ELISA technique. Viral detection and typing were done by PCR for adenovirus, and by Reverse transcriptase PCR for rotavirus, astrovirus and norovirus.

**RESULTS:**

Serum I-FABPs level was significantly higher in the cases compared to the controls and was also higher in the 46 rotavirus gastroenteritis cases compared to other viral gastroenteritis cases. Serum I- FABPs level was significantly higher in severely dehydrated cases as compared to mildly dehydrated ones (P=0.037).

**CONCLUSION:**

Serum I-FABPs could be used as an early and sensitive predictor marker of gut wall integrity loss in children with viral gastroenteritis and its level can indicate case severity.

## Introduction

Acute viral gastroenteritis (GE) is one of the commonest causes of morbidity and mortality in very young aged children [1-3]. This is definitely due to the dehydrating diarrhea and might be also due to secondary blood stream infection [4]. Four major viral pathogens associated with AGE are three RNA viruses (rotavirus, norovirus, and astrovirus) and one DNA virus (enteric adenovirus) [4-6]. Co-infections with these viruses have been frequently reported especially rotavirus and norovirus co-infections [7-9].

Rotavirus remains the most common cause of severe childhood diarrhea worldwide and of diarrheal mortality in developing countries [10]. The World Health Organization (WHO) estimates that 527,000 children under the age of five years die of rotavirus disease each year [11]. Children in the poorest countries account for 82% of rotavirus deaths in children, fewer than five years of age [12, 13].

Loss of gut wall integrity, and intestinal ischemia are consequence of low flow states associated with viral gastroenteritis. Prolonged periods of intestinal ischemia reperfusion result in severe damage to the intestinal barrier, thereby allowing translocation of bacteria from the intestinal lumen to the systemic circulation [14]. As early detection of intestinal ischemia reperfusion is essential to improve clinical outcome, Derikx et al., [15], and Grootjans et al., [16] have studied the diagnostic value of biomarkers to detect enterocyte membrane integrity loss, an early phenomenon during intestinal ischemia.

Intestinal fatty-acid binding protein (I-FABP) is a newly sensitive plasma marker of for early enterocyte membrane integrity loss. Several studies have described the use of I-FABP for the early detection of intestinal injury due to decreased perfusion of the small bowel, inflammation, or intestinal ischemia [17, 18]. It is part of a family of nine different FABP types, each named after the tissue of its first detection. I-FABPs are small (15 kDa) low molecular weight cytosolic proteins specifically present in mature enterocytes at the tip of the villus of small intestine and constitute 2% of enterocyte proteins. They are released upon enterocyte membrane integrity loss into the circulation, which makes them useful as plasma markers for enterocyte damage [19]. Assessment of gut wall integrity in clinical practice is still a challenge, as it is difficult to evaluate the condition of the gut non-invasively with currently available diagnostic tools. Moreover, non-invasive, rapid diagnostic means to assess intestinal condition are needed to evaluate the effects of treatment of intestinal disorders. Therefore, the aim of this current study is to assess serum intestinal fatty acid binding protein (I-FABP) as an early and sensitive predictor for the evaluation of gut wall integrity loss in GE particularly rotavirus gastroenteritis (RV-GE) in Egypt.

## Subjects and Methods

### Subjects

This cross sectional case control study was conducted on 93 cases of acute gastroenteritis. They were 55 males and 38 females. Their ages ranged from newborn up to 5 years old. They belonged to different social classes and were taken from Cairo, Giza and Kalyoubia governorates in Egypt. Thirty-two of these cases were hospitalized while the other sixty-one cases were not. Of these, eleven cases just entered the hospital to receive I.V. fluids and then were dismissed. The remaining 50 cases were taken from the outpatient clinics of the same hospitals.

### Inclusion Criteria

1. Age range (newborn up to 5 years old).
2. Boys and girls of the same social classes are included.

### Exclusion Criteria

1. Infants receiving Rota virus vaccine.
2. Children on corticosteroids or immunosuppressive drug therapy.
3. Children complaining of systemic auto-immune disease, immunodeficiency, chronic renal disease, or cancer.
4. Previous blood or any blood products transfusion.
5. History of chronic allergic diseases.

The control group comprised 28 apparently healthy children who did not have diarrhea during the 2 weeks period preceding enrollment in the study. They belonged to the same regions and were of the same age range. They were 13 males and 15 females.

Ethical approval was obtained from the Medical Ethical Committee of the National Research Centre. Written informed consent was obtained from the parents after explanation of the aim of the study and its possible benefits for identifying the cause of the acute viral gastroenteritis of their children.

### Methods

All cases were subjected to full history taking, thorough clinical examination, and collection of clinical data. Clinical data included disease manifestations as fever, vomiting, abdominal pain or bloody diarrhea. Severity criteria like duration of the diarrhea, number of stool motions or bouts of vomiting, range of body temperature, and degree of dehydration were determined for all hospitalized children.

Blood samples approximately 3 ml were taken from all cases and controls for measuring intestinal fatty acid binding protein (I-FABP). Serum was immediately separated from each blood sample and stored frozen at −20°C till the measurement of I-FABP. Serum I-FABP levels were analyzed using the human I-FABP-specific enzyme-linked immunos-orbent assay (ELISA) commercial test kit (EIAab^®^, China), according to the following steps. Firstly, 100 μl of standard, blank, and samples were added per well that wells were pre-coated with biotin-conjugated polyclonal antibody specific to human iFABP. Then, the plate covered with the plate sealer was incubated for 2 hours at 37°C. After incubation 100 μl of avidin conjugated was added to each well then plate covered and incubated for 1 hour at 37°C then each well aspirated and washed three times. After washing, 100 μl of horseradish peroxidase (HRP) was added to each microplate well then the plate incubated for 1 hour at 37°C. After incubation each well was aspirated and washed. This wash step was followed by addition of 90 μl TMB substrate solution to each well then the plate incubated for 30 minutes at 37°C and protected from light. The enzyme-substrate reaction was terminated by the addition of 50 μl sulphuric acid solution and the color change was measured using microplate reader at a wavelength of 450 nm [20].

Stool analysis was done by using Polymerase Chain Reaction (PCR) and by Reverse transcriptase PCR (RT-PCR) for viral detection and typing. Stool samples were collected in dry sterile clean plastic cups. Collected samples were diluted and prepared for PCR, and RT-PCR for detection of viral gastroenteritis agents in the collected samples. Viral gastroenteritis was detected in all collected stool samples were subjected to extraction of both viral RNA and DNA in the samples using Axygen^®^ Kit (Axygen biosciences, Cat. No AP-MN-BF-VNA-250) to facilitate the detection of both RNA gastroenteritis viruses (such as rotaviruses, noroviruses, and astroviruses) and DNA gastroenteritis viruses (such as adenoviruses).

Viral genomes were extracted from 10% diluted stool samples using Axygen^®^ Kit (Axygen biosciences, Cat. No. AP-MN-BF-VNA-250) according to manufacturers instructions and prepared for detection of viral gastroenteritis by both RT-PCR and PCR using sets of oligonucleotide primers specific to each type of virus Universal viral gastroenteritis oligonucleotide primers sequences were obtained from Bioneer Company, as purified lyophilized primers. Universal primers for G typing rotaviruses were selected for amplification of segment 9 as previously described [21]. These primer pairs are semi nested primers designed to amplify the highly conserved region among all known rotaviruses strains and specific for gene segment 9, as. Universal primers for P typing rotaviruses were selected for amplification of VP7 gene segment [22]. For detection of astroviruses sets of primers were selected and used as described by [23], and noroviruses sets of primers were used [24]. For adenoviruses PCR the primer sets described by [25] and designed to amplify the highly conserved region of hexon gene which is very similar in many types of adenoviruses.

Reverse transcriptase-polymerase chain reaction (RT-PCR):

All collected stool samples were subjected to extraction of both viral RNA and DNA in the samples to facilitate the detection of both RNA gastroenteritis viruses such as rotaviruses, noroviruses, and astroviruses and DNA gastroenteritis viruses such as adenoviruses. Viral genomes were extracted from 10% diluted stool samples and prepared for detection of viral gastroenterities by both RT-PCR and PCR using sets of oligonucleotide primers specific to each type of virus as follow:

### Rotaviruses

cDNA synthesis and PCR amplification: Viral RNA was extracted from 200 µl aliquot of 10% stool sample, using Axygen^®^ Kit (Axygen biosciences, Cat. No. AP-MN-BF-VNA-250), according to the instruc-tions of the supplier. Reverse transcription (RT) was carried out using a mixture of 10 µl of extracted viral RNA, 5 µl random primer (25 pmol/µl), and 20 µl DEPC treated water. The reaction mixture, 35 µl, was heated at 65ºC for 5 min. and chilled on ice for 2 min. 35 µl of the reaction mixture was added to 1µl of 200 U/µl M-MLV Reverse Transcriptase (Promega, Cat. # M1701), 10 µl RT-buffer, 4 µl of 10mM each dNTPs (Promega, Cat. # U1511). The mixture was heated at 42ºC for 60 min and 37ºC for 30 min, for cDNA synthesis, followed by 95ºC for 5 min. Nested RT-PCR the reaction takes place in two rounds; of The first-round PCR was carried out in 50μl reaction mixture containing 10 μl of the synthesized cDNA, 5 μl 10 x PCR Buffer, 4 μl of 25 mM MgCl_2_ (Promega, Cat. # A3511), 0.5 μl of 5 U/μl Go Taq DNA Polymerase (Promega, Cat. # M8305), 1 μl (50 pmol/μl) of sense primer, 1 μl (50 pmol/μl) of antisense primer, 4 μl of 10 mM each dNTPs (Promega, Cat. # U1511) and 24.5 μl DEPC treated water. For PCR amplification of G type rotaviruses (Table 1), an initial denaturation of 5min at 95ºC was applied and 35 cycles for first round and 30 cycles for 2nd round were used. The PCR profile for first round was 94ºC for 1 min, 52ºC for 1 min, and 72ºC for 1 min, and an additional extension at 72ºC for 7min was applied using a PCR machine (BioMetra, UK). The amplified RT-PCR products were electrophoresed through 1.5% agarose gels in Tris acetate/EDTA buffer against 100 bp DNA ladder (Promega, Cat. # G2101) and visualized by staining with ethidium bromide. Bands were visualized under UV light using UV transilluminator. The obtained band size is determined with the reference of the DNA ladder.

**Table 1:** PCR reaction profile for G type Rotaviruses.

Step	Reaction			

	1st round		2nd round	

	Temp (°C)	Time (min)	Temp (°C)	Time (min)
Denaturation	94	1 min	94	1 min

Annealing	52	1 min	42	2 min

Extension	72	1 min	72	1 min

No. of cycles	35		30	

Expected Product	881 bp		175-754 bp	

For the second round; PCR amplification was carried out from the first round PCR reaction in 50μl reaction mixture containing 2 μl of 1st round PCR product, 5 μl 10 x PCR Buffer, 4 μl of 25 mM MgCl_2_ (Promega, Cat. # A3511), 0.5 μl of 5 U/μl Go Taq Flexi DNA Polymerase (Promega Cat. No. M8301), 1 μl downstream primer (50 pmol/μl), 1 μl of 8 cocktail upstream primers (50 pmol/μl each), 4 μl of 10 mM each dNTPs (Promega, Cat. # U1511) and 25.5 μl DEPC treated water. The PCR profile for the second round was an initial denaturation of 5 min at 95ºC followed by 30 cycle at 94ºC for 1 min, 42ºC for 2 min, and 72ºC for 1 min, and an additional extension at 72ºC for 7 min was applied using a PCR machine (BioMetra, UK). The amplified RT-PCR products were electrophoresed through 1.5% agarose gels in Tris acetate/EDTA buffer against 100bp DNA ladder (Promega, Cat. # G2101) and visualized by staining with ethidium bromide. Bands were visualized under UV light using UV transilluminator. The obtained band size is determined with the reference of the DNA ladder.

For PCR amplification of P type rotaviruses (Table 2), an initial denaturation of 5min at 95ºC and 35 cycles for first round and 30 cycles for 2nd round. For 1st round PCR cycles profiles was at 94ºC for 1min, 50ºC for 2 min, and 72ºC for 1 min, and an additional extension at 72ºC for 7 min. Profile was applied using a PCR machine (BioMetra, UK). The amplified RT-PCR products were electrophoresed through 1.5% agarose gels in Tris acetate/EDTA buffer against 100bp DNA ladder (Promega, Cat. # G2101) and visualized by staining with ethidium bromide. Bands were visualized under UV light using UV transilluminator. The obtained band size is determined with the reference of the DNA ladder.

**Table 2:** PCR reaction profile for P type Rotaviruses.

Step	Reaction			

	1st round		2nd round	

	Temp (°C)	Time (min)	Temp (°C)	Time (min)
Denaturation	94	1 min	94	1 min

Annealing	50	2 min	45	2 min

Extension	72	1 min	72	1 min

No. of cycles	35		30	

Expected Product	876 bp		276-583bp	

For the second round; PCR amplification was carried out from the first round PCR reaction in 50 μl reaction mixture containing 2 μl of 1st round PCR product, 5μl 10x PCR Buffer, 4 μl of 25 mM MgCl_2_ (Promega, Cat. # A3511), 0.5 μl of 5 U/μl Go Taq Flexi DNA Polymerase (Promega Cat. No. M8301), 1 μl of a 7 cocktail downstream primers (50 pmol/μl each), 1 μl upstream primer (50 pmol/μl), 4 μl of 10 mM each dNTPs (Promega, Cat. # U1511) and 26.5 μl DEPC treated water. The PCR profile for the second round was an initial denaturation of 5min at 95ºC followed by 30 cycle at 94ºC for 1 min, 45ºC for 2 min, and 72ºC for 1 min, and an additional extension at 72ºC for 7 min was applied using a PCR machine (BioMetra, UK). The amplified RT-PCR products were electrophoresed through 1.5% agarose gels in Tris acetate/EDTA buffer against 100bp DNA ladder (Promega, Cat. # G2101) and visualized by staining with ethidium bromide. Bands were visualized under UV light using UV transilluminator. The obtained band size is determined with the reference of the DNA ladder.

### Astroviruses

cDNA synthesis and PCR amplification: Viral RNA was extracted from 200 µl aliquot of 10% stool sample, using Axygen^®^ Kit (Axygen biosciences, Cat. No. AP-MN-BF-VNA-250), according to the instructions of the manufacturer. Reverse transcription (RT) was carried out using a mixture of 10 µl of extracted viral RNA, 5 µl random primer (25 pmol/µl), and 20 µl DEPC treated water. The reaction mixture, 35 µl, was heated at 65ºC for 5 min and chilled on ice for 2 min 35 µl of the reaction mixture was added to 1 µl of 200 U/µl M-MLV Reverse Transcriptase (Promega, Cat. # M1701), 10 µl RT-buffer, 4 µl of 10 mM each dNTPs (Promega, Cat. # U1511). The mixture was heated at 42ºC for 60 min and 37ºC for 30 min, for cDNA synthesis, followed by 95ºC for 5 min. The RT-PCR reaction was carried out in 50 μl reaction mixture containing 10 μl of the synthesized cDNA, 5 μl 10 x PCR Buffer, 4 μl of 25 mM MgCl_2_ (Promega, Cat. # A3511), 0.5 μl of 5 U/μl Go Taq DNA Polymerase (Promega, Cat. # M8305), 1 μl (50 pmol/μl) of sense primer, 1μl (50 pmol/μl) of antisense primer, 4 μl of 10 mM each dNTPs (Promega, Cat. # U1511) and 24.5 μl DEPC treated water. For PCR amplification for astroviruses (Table 3), was start by an initial denaturation of 5min at 95ºC, followed by 40 thermal cycles. The PCR profile for thermal cycles was 94ºC for 1 min, 50ºC for 1 min, and 72ºC for 1 min, and an additional extension at 72ºC for 7 min was applied using a PCR machine (BioMetra, UK). The amplified RT-PCR products were electrophoresed through 1.5% agarose gels in Tris acetate/EDTA buffer against 100 bp DNA ladder (Promega, Cat. # G2101) and visualized by staining with ethidium bromide. Bands were visualized under UV light using UV transilluminator. The obtained band size is determined with the reference of the DNA ladder.

**Table 3:** PCR reaction profile for Astroviruses.

Step	Reaction	

	Temp (°C)	Time (min)
Denaturation	94	1 min

Annealing	50	2 min

Extension	72	1 min

No. of cycles	40	

Expected Product	449 bp	

### Noroviruses genogroup I (GI)

cDNA synthesis and PCR amplification: Viral RNA was extracted from 200 µl aliquot of 10% stool sample, using Axygen^®^ Kit (Axygen biosciences, Cat. No. AP-MN-BF-VNA-250), according to the instructions of the manufacturer. Reverse transcription (RT) was carried out using a mixture of 10 µl of extracted viral RNA, 5 µl random primer (25 pmol/µl), and 20 µl DEPC treated water. The reaction mixture, 35 µl, was heated at 65ºC for 5 min. and chilled on ice for 2 min. 35 µl of the reaction mixture was added to 1 µl of 200 U/µl M-MLV Reverse Transcriptase (Promega, Cat. # M1701), 10 µl RT-buffer, 4 µl of 10mM each dNTPs (Promega, Cat. # U1511). The mixture was heated at 42ºC for 60 min and 37ºC for 30 min, for cDNA synthesis, followed by 95ºC for 5 min. The RT-PCR reaction was carried out in 50 μl reaction mixture containing 10 μl of the synthesized cDNA, 5 μl 10 x PCR Buffer, 4 μl of 25 mM MgCl_2_ (Promega, Cat. # A3511), 0.5μl of 5U/μl Go Taq DNA Polymerase (Promega, Cat. # M8305), 1 μl (50 pmol/μl) of sense primer, 1 μl (50 pmol/μl) of antisense primer, 4 μl of 10mM each dNTPs (Promega, Cat. # U1511) and 24.5 μl DEPC treated water. For PCR amplification for Noroviruses GI (Table 4), the reaction started by an initial denaturation of 5 min at 95ºC, followed by 40 thermal cycles. The PCR profile for thermal cycles was 94ºC for 45 sec, 50ºC for 45 sec, and 72ºC for 1 min, and an additional extension at 72ºC for 7 min was applied using a PCR machine (BioMetra, UK). The amplified RT-PCR products were electrophoresed through 1.5% agarose gels in Tris acetate/EDTA buffer against 100 bp DNA ladder (Promega, Cat. # G2101) and visualized by staining with ethidium bromide. Bands were visualized under UV light using UV transilluminator. The obtained band size is determined with the reference of the DNA ladder.

**Table 4:** PCR reaction profile for Noroviruses GI.

Step	Reaction	

	Temp (°C)	Time (min)
Denaturation	94	45 sec

Annealing	50	45 sec

Extension	72	1 min

No. of cycles	40	

Expected Product	329 bp	

### Noroviruses genogroup II (GII)

cDNA synthesis and PCR amplification: Viral RNA was extracted from 200 µl aliquot of 10% stool sample, using Axygen^®^ Kit (Axygen biosciences, Cat. No. AP-MN-BF-VNA-250), according to the instructions of the manufacturer. Reverse transcription (RT) was carried out using a mixture of 10 µl of extracted viral RNA, 5 µl random primer (25 pmol/µl), and 20 µl DEPC treated water. The reaction mixture, 35 µl, was heated at 65ºC for 5 min and chilled on ice for 2 min. 35µl of the reaction mixture was added to 1µl of 200 U/µl M-MLV Reverse Transcriptase (Promega, Cat. # M1701), 10 µl RT-buffer, 4 µl of 10mM each dNTPs (Promega, Cat. # U1511). The mixture was heated at 42ºC for 60 min and 37ºC for 30 min, for cDNA synthesis, followed by 95ºC for 5 min. The RT-PCR reaction was carried out in 50 μl reaction mixture containing 10 μl of the synthesized cDNA, 5 μl 10 x PCR Buffer, 4 μl of 25 mM MgCl_2_ (Promega, Cat. # A3511), 0.5 μl of 5 U/μl Go Taq DNA Polymerase (Promega, Cat. # M8305), 1 μl (50 pmol/μl) of sense primer, 1 μl (50 pmol/μl) of antisense primer, 4 μl of 10 mM each dNTPs (Promega, Cat. # U1511) and 24.5 μl DEPC treated water. For PCR amplification for Noroviruses GII (Table 5), the reaction started by an initial denaturation of 5min at 95ºC, followed by 40 thermal cycles. The PCR profile for thermal cycles was 94ºC for 30 sec, 50ºC for 30 sec, and 72ºC for 1 min, and an additional extension at 72ºC for 7 min was applied using a PCR machine (BioMetra, UK). The amplified RT-PCR products were electrophoresed through 1.5% agarose gels in Tris acetate/EDTA buffer against 100 bp DNA ladder (Promega, Cat. # G2101) and visualized by staining with ethidium bromide. Bands were visualized under UV light using UV transilluminator. The obtained band size is determined with the reference of the DNA ladder.

**Table 5:** PCR reaction profile for Noroviruses GII.

Step	Reaction	

	Temp (°C)	Time (min)
Denaturation	94	30 sec

Annealing	50	30 sec

Extension	72	1 min

No. of cycles	40	

Expected Product	343 bp	

### DNA Viruses (Adenoviruses)

PCR amplification: Viral DNA was extracted from 200µl aliquot of 10% diluted stool samples, with sterile PBS pH 7.2, using Axygen^®^ Kit, (Axygen biosciences, Cat. No. AP-MN-BF-VNA-250), according to the instructions of the manfacturer. Nested PCR was carried out where the reaction takes place in two rounds; of The first-round PCR was carried out in 50 μl reaction mixture containing 10 μl of extracted DNA, 5 μl 10 x PCR Buffer, 4 μl of 25 mM MgCl_2_ (Promega, Cat. # A3511), 0.5 μl of 5 U/μl Go Taq DNA Polymerase (Promega, Cat. # M8305), 1 μl (50 pmol/μl) of sense primer (Adv-HEX1DEG-F10), 1 μl (50 pmol/μl) of antisense primer (Adv-HEX2DEG-R10), 4 μl of 10 mM each dNTPs (Promega, Cat. # U1511) and 24.5 μl DEPC treated water. For PCR amplification of adenoviruses (Table 6), an initial denaturation of 5 min at 95ºC was applied and 35 cycles for first round and 35 cycles for 2nd round were used. The PCR profile for first round was 94ºC for 30 sec, 55ºC for 30 sec, and 72ºC for 1 min, and an additional extension at 72ºC for 7 min was applied using a PCR machine (BioMetra, UK). The amplified PCR products were electrophoresed through 1.5% agarose gels in Tris acetate/EDTA buffer against 100 bp DNA ladder (Promega, Cat. # G2101) and visualized by staining with ethidium bromide. Bands were visualized under UV light using UV transilluminator. The obtained band size is determined with the reference of the DNA ladder.

**Table 6:** PCR reaction profile for Adenoviruses.

Step	Reaction			

	1st round		2nd round	

	Temp (°C)	Time (min)	Temp (°C)	Time (min)
Denaturation	94	30 sec	94	30 sec

Annealing	55	30 sec	55	30 sec

Extension	72	1 min	72	1 min

No. of cycles	35		35	

Expected Product	301 bp		171 bp	

For the second round; PCR amplification was carried out from the first round PCR reaction in 50 μl reaction mixture containing 5 μl of 1st round PCR product, 5 μl 10 x PCR Buffer, 4 μl of 25 mM MgCl_2_ (Promega, Cat. # A3511), 0.5μl of 5U/μl Go Taq Flexi DNA Polymerase (Promega Cat. No. M8301), 1 μl of (Adv-HEX3DEG-F10) downstream primer (50 pmol/μl), 1 μl of (Adv-HEX4DEG-R10) upstream primers (50 pmol/μl each), 4 μl of 10 mM each dNTPs (Promega, Cat. # U1511) and 28.5 μl DEPC treated water. The PCR profile for the second round was an initial denaturation of 5 min at 95ºC followed by 35 cycle at 94ºC for 30 sec, 55ºC for 30 sec, and 72ºC for 1 min, and an additional extension at 72ºC for 7 min was applied using a PCR machine (BioMetra, UK). The amplified PCR products were electrophoresed through 1.5% agarose gels in Tris acetate/EDTA buffer against 100 bp DNA ladder (Promega, Cat. # G2101) and visualized by staining with ethidium bromide. Bands were visualized under UV light using UV transilluminator. The obtained band size is determined with the reference of the DNA ladder.

### Oligonucleotide primers

Universal viral gastroenteritis oligonucleotide primers sequences were obtained from Bioneer Company, as purified lyophilized primers. Universal primers for G typing rotaviruses was selected for amplification of segment 9 as previously described by Iturriza-Gomara, et al., [21]. These primer pairs are semi nested primers designed to amplify the highly conserved region among all known rotaviruses strains and specific for gene segment 9, as listed and shown in Table 7. Universal primers for P typing rotaviruses was selected for amplification of VP7 gene segment as described by Gentsch et al., [22] and also listed and shown in Table 7. For detection of astroviruses sets of primers were selected and used as described by Noel et al., [23] (Table 7), and noroviruses sets of primers were used as described by Kojima et al., [24] (Table 7). For adenoviruses PCR the primer sets listed in Table 7, described by Allard et al., [25] and designed to amplify the highly conserved region of hexon gene which is very similar in many types of adenoviruses.

**Table 7:** The list of primers sets used to detect viruses.

Primer	Sequence	Polarity	Location
**G genotype of rotaviruses**			
1st round			
Rota VP7-F10	ATGTATGGTATTGAATATTACCAC	+	51
Rota VP7-R10	AACTTGCCACCATTTTTTCC	-	932
2nd round			
Rota G1-aBT1-10	CAAGTACTCAAATCAATGATGG	+	314
Rota G2-acT2-10	CAATGATATTAACACATTTTCTGTG	+	411
Rota G- 10	ACGAACTCAACACGAGAGG	+	250
Rota G4-2aDT4-10	CGTTTCTGGTGAGGAGTTG	+	480
Rota G8-aAT8-10	GTCACACCATTTGTAAATTCG	+	178
Rota G9-10	CTTGATGGACTAYAAATAC	+	757
Rota G10-10	ATGTCAGACTACARATACTG	+	666
Rota G9-VP7aFT9d-10	CTTGATGTRACTAYAAMTAC	+	757
Rota VP7-R10	AACTTGCCACCATTTTTTCC	-	932
**P genotype of rotaviruses**			
1st round:			
Con3-10	TGGCTTCGCCATTTTATAGA	+	11
Con2-10	ATTTCGGACCATTTATAACC	-	885
2nd round:			
Con3-10	TGGCTTCGCCATTTTATAGA	+	11
Rota P(8)-1T1-10	TCTACTTGGATAACGTGC	-	356
Rota P(4)-2T1-10	CTATTGTTAGAGGTTAGAGTC	-	494
Rota P(6)-3T1-10	TGTTGATTAGTTGGATTCAA	-	278
Rota P(9)-4T1-10	TGAGACATGCAATTGGAC	-	402
Rota P(10)-5T1-10	ATCATAGTTAGTAGTCGG	-	594
Rota P(11) -10	GTAAACATCCAGAATGTG	-	323
Rota P(8)-1T-1D-10	TCTACTGGRTTRACNTGC	-	356
**Astroviruses**			
Astro-mon-269-F-10	CAACTCAGGAAACAGGGTGT	+	4526
Astro-mon-270-R-10	TTAGTGAGCCACCAGCCATC	-	4974
**Noroviruses Genogroup I (GI)**			
Noro GISKF-10	CTGCCCGAATTYGTAAATGA	+	5342
Noro GISKR-10	CCAACCCARCCATTRTACA	-	5671
**Noroviruses Genogroup II (GII)**			
Noro GII SKF-10	CNTGGGAGGGCGATCGCAA	+	5058
Noro GII SKF-10	CCRCCNGCATRHCCRTTRTACAT	-	5401
**Adenoviruses**			
1^st^ round			
Adv-HEX1 DEG10	GCCSCARTGGKCWTACATGCACATC	+	21
Adv-HEX 2 DEG10	CAGCACSCCICGRATGTCAAA	-	321
2^nd^ round			
Adv-HEX3 DEG- F10	GCCCGYGCMACIGAIACSTACTTC	+	68
Adv-HEX4 DEG-R1	CCYACRGCCAGIGTRWAICGMRCYTTGTA	-	239

### Analysis of PCR products

PCR products were analyzed by electrophoresis using 1.5% Agarose gel with Ethidium bromide, as follow: 1.5 g umltrapure Agarose powder (BDH), was dissolved in 100 ml Tris-Borate-EDTA (TBE) and cooked at microwave for 2 min. Agarose was cooled down to about 42-45°C and 8 µl of Ethidium bromide (10 mg/ml) was added. The melted agarose was poured into horizontal electrophoresis chamber with 20 whole comb and left to dry at room temperature. 10 µl of PCR products were mixed with 2 µl of loading buffer (1% sodium dodecyl sulphate, 0.25% bromophenol blue and 0.1M EDTA, pH 8.0), and applied into agarose gel and electrophoresed at 100V for 1-1.5 hr. Presumptive and confirmed product bands were visualized and compared with standard 100 bp DNA marker (Promega, USA, Cat. # G2101). Bands were visualized under UV light using UV transilluminator and gel documentation system.

### Statistical analysis

Statistical analysis was performed using the SPSS statistical package software for windows version 21 (SSPS Inc, Pennsylvania, and USA). Parametric variables are expressed as the mean ± SD. Differences between parametric variables among the obese and overweight versus control groups were evaluated using 2-tailed unpaired t-test. Pearson’s correlation coefficients were used to evaluate correlations between the data exhibiting parametric distribution. P value <0.05 was considered significant difference and p < 0.005 was considered highly significant difference.

## Results

Serum levels of I-FABP were higher in GE cases group compared to control group (1026.4 pg/ml vs 267.9 pg/ml, respectively). Table 8 showed serum I-FABP level in gastroenteritis cases group versus control group. Serum levels of I-FABP were higher in the 46 Rotavirus - GE cases compared to the 9 adenovirus – GE cases. Also these levels were higher in the 46 Rotavirus- GE cases compared to the 4 Astrovirus-GE cases. There were 3 cases with mixed Rotavirus and Adenovirus co-infections whose serum I-FABP levels shouted to 1425 pg/ml. Similarly, the other 3 cases of mixed Rotavirus and Astrovirus co-infections had those levels reaching 1217 pg/ml. Although those cases were few in number but their importance will be stated in the discussion.

**Table 8:** Serum I-FABP level in gastroenteritis cases group versus control group.

Serum I-FABP conc. (pg/ml)	Number	Mean ± SD	P- value
GE cases	93	1026.4 ± 494.4	P<0.001
Control	28	267.9 ± 200.4	P<0.001

**highly significant difference at p<0.001.

Table 9 showed the serum I-FABP level according to type of virus.

**Table 9:** Serum I-FABP level according to type of virus.

Variable	Rotavirus cases	Adenovirus cases	Astrovirus cases	Rotavirus and Adenovirus cases	Rotavirus and Astrovirus cases	Total GE cases	Control
No. of cases	46	9	4	3	3	93	28

Serum I-FABP	1017.4 ± 456*	653 ± 419	408 ± 210	1425 ± 871	1217 ± 453	1217 ± 453	267.9 ± 200.4*

* Significant difference at p<0.05.

Paired Samples Test revealed statistically significant difference in I-FABP level when mild and severe degrees of dehydration were compared. On the other hand, statistically insignificant difference in I-FABP level between mild and moderate degrees of dehydration is observed in Table 10.

**Table 10:** Comparisons between serum I-FABP levels according to degree of dehydration in the studied groups.

Serum I-FABP level	t	P value
Mild vs Moderate cases	0.318	0.760

Mild vs Severe cases	2.564	0.037*

* Significant difference at p<0.05.

Significant positive correlations were found between hospitalization and serum level of I-FABP as well as dehydration (P=0.000). Furthermore, a significant positive correlation was found out between hospitalization and the degree of fever (P=0.002) as shown in Table 11.

**Table 11:** Correlations between hospitalization and other parameters in the study group.

Hospitalization		Serum I-FABP	Dehydration severity	Fever

	Pearson correlation	0.223*	0.713**	0.322**

	Sig. (2-tailed)	0.045	0.000	0.002

* Significant difference at p<0.05,

** highly significant difference at p<0.001.

## Discussion

Loss of gut wall integrity is consequence of low flow states associated with viral gastroenteritis. It is involved in the development of various inflammatory syndromes, including sepsis, and multiple organ failure. The delay in diagnosis is a major factor associated with the high morbidity and mortality of patients with intestinal ischemia [26]. Although diagnostic accuracy of intestinal ischemia has improved with the development of computed tomography (CT) [27] a laboratory test for clinical use is necessary [28]. Intestinal fatty acid–binding protein (I-FABP) is a useful marker in the detection of intestinal ischemia. These are mainly expressed in the intestinal villi which are the initial site of destruction in acute intestinal inflammation, injury, and ischemia all of which are present in viral gastroenteritis pathophysiology [29]. It has emerged as a valuable marker in the diagnosis of intestinal damage at an early stage [30, 31]. The current study provides additional information concerning the clinical utility of serum I-FABP levels during intestinal ischemia reperfusion.

Our present study dealt with the promising issue of resorting to serum I-FABPs in the early prediction of intestinal injury in viral GE particularly Rota viral GE. This is very crucial to Egypt and other developing countries because Rota virus vaccination is still fighting against poor sanitation in general and the sewage polluted River-Nile water in particular. Our study worked upon proving that, I-FABP could be made use as early predictor of the gut injury in viral GE.

Several studies showed that plasma I-FABP levels were significantly increased in patients suspected with intestinal ischemia, as compared with patients with other diagnoses [32]. It increases within the early stages of intestinal ischemia [33]. In our present study, serum I-FABP levels were significantly higher in GE cases compared to control group.

Many recent studies have also reported the existence of coinfections by different viruses whether in Egypt or in other countries [34-36]. Some studies have confirmed that in RV-GE cases, viral co-infections (and even bacterial and parasitic) aggravated the clinical picture of the disease and caused an extension of the hospital stay period [37, 38]. Some recent studies reported the predominance of Adenovirus and Astrovirus GE in certain countries in particular months of the year [39-40]. In our present study, we did have serious conditions which necessitated hospitalization, and serum I-FABPs levels were also higher in RV-GE cases compared to Adeno and Astro viruses GE cases. In addition, their levels were also higher in GE cases infected with mixed viruses (coinfections). Similar to our results, Ishimura et al., [41], and Pelsers et al., [42] found an elevation in serum levels of I-FABP in cases of intestinal inflammation, injury and ischemia. In addition, their levels were not detectable in serum of healthy subjects.

Guthmann et al., [17] ensured that, I-FABP was a very specific marker for detection of intestinal injury which led to severe stage III. In accordance with our results, some studies emphasized that individuals who suffered from necrotizing enterocolitis (NEC), and acute ischemic diseases had their serum I-FABP concentrations applicable for detection of intestinal injury [20]. Again, in agreement with our study, Panl et al., [43] assumed that I-FABP might constitute a useful marker for acute intestinal failure in critically ill patients.

Machado et al., [36] found out that levels of I-FABP were early marker of intestinal necrosis after acute pancreatitis and after liver surgery and aortic surgery. In this study, critically ill patients had an evaluation of their intestinal failure via evaluating the circulating I-FABP values. Many studies also described I-FABP as plasma markers for intestinal injury [42, 43]. Although they are relatively old ones, still very important because all those research studies agreed upon that finding and the rarity of similar recent studies does not be little the crucial importance of old ones.

In our study, we observed a significant elevation in serum I-FABP level on comparing severely dehydrated groups with mild dehydrated cases. There was a positive correlation between the serum level of I-FABP and hospitalized patients, taking into consideration that severely dehydrated children needed hospitalization.

In conclusions, this study provides new insight into the significance of serum I-FABP following human intestinal injury. The results demonstrate that the extent of intestinal tissue damage is related to the duration of injury. In addition, systemic I-FABP levels can very well differentiate between mild, reversible damage and more extensive irreversible intestinal damage. Future studies should be conducted using I-FABP in the decision-making during the diagnostic workup of patients suspected for intestinal injury, to integrate I-FABP in daily clinical practice.
